# Innate and Adaptive Immune Interactions at the Fetal–Maternal Interface in Healthy Human Pregnancy and Pre-Eclampsia

**DOI:** 10.3389/fimmu.2014.00125

**Published:** 2014-03-28

**Authors:** Peter Hsu, Ralph Kay Heinrich Nanan

**Affiliations:** ^1^Charles Perkins Centre Nepean, Penrith, NSW, Australia; ^2^Department of Allergy and Immunology, The Children’s Hospital at Westmead, Sydney, NSW, Australia; ^3^Sydney Medical School, The University of Sydney, Sydney, NSW, Australia

**Keywords:** pregnancy, pre-eclampsia, T regulatory cells, decidual, NK cells, CD4^+^HLA-G^+^, dendritic cells

## Abstract

Maternal immune tolerance of the fetus is indispensable for a healthy pregnancy outcome. Nowhere is this immune tolerance more important than at the fetal–maternal interface – the decidua, the site of implantation, and placentation. Indeed, many lines of evidence suggest an immunological origin to the common pregnancy-related disorder, pre-eclampsia. Within the innate immune system, decidual NK cells and antigen presenting cells (including dendritic cells and macrophages) make up a large proportion of the decidual leukocyte population, and are thought to modulate vascular remodeling and trophoblast invasion. On the other hand, within the adaptive immune system, Foxp3^+^ regulatory T cells are crucial for ensuring immune tolerance toward the semi-allogeneic fetus. Additionally, another population of CD4^+^HLA-G^+^ suppressor T cells has also been identified as a potential player in the maintenance of immune tolerance. More recently, studies are beginning to unravel the potential interactions between the innate and the adaptive immune system within the decidua, that are required to maintain a healthy pregnancy. In this review, we discuss the recent advances exploring the complex crosstalk between the innate and the adaptive immune system during human pregnancy.

## Introduction

Pregnancy presents a significant challenge to the maternal immune system. In humans, the maternal immune system must tolerate the semi-allogeneic fetus throughout the 9 months of pregnancy. The remarkable nature of this phenomenon was recognized by Peter Medawar in the 1950s (1), whose work on skin graft rejection in genetically different individuals, led him to perceive this apparent immunological paradox. At the time, he proposed that three factors contribute to this phenomenon: (1) the anatomical separation between the mother and the fetus, (2) the reduced antigenic property of the fetus, and (3) the immunological inertness of the maternal immune system.

These proposals have significantly influenced subsequent research in the field. Indeed, it is now well-known that fetal cells are largely separated from the maternal immune system, with the point of contact being fetal extravillous trophoblast (EVT) cells, which have poor antigenic properties owing to the lack of expression of classical MHC class I (except HLA-C) and MHC class II molecules (2). However, as fetal–maternal microchimerism is a well-recognized occurrence during human pregnancy, fetal cells frequently induce maternal immune activation (3, 4) as evidenced by the detection of anti-fetal HLA antibodies in maternal serum during pregnancy (5, 6). Additionally, although direct MHC presentation of fetal antigens by fetal cells generally does not occur, fetal antigens can be processed and presented by maternal antigen presenting cells (APCs) at the fetal–maternal interface (7). Indeed, various different subsets of maternal immune cells are present at the fetal–maternal interface, which is the decidua, the mucous membrane (endometrium) of the pregnant uterus. In fact, up to 50% of the cells in the decidua are maternal immune cells (8). The decidua is therefore, an important site where the maternal immune system encounters fetal antigens and must develop tolerance mechanisms.

Not surprisingly, many of the pregnancy-related disorders such as recurrent miscarriages and pre-eclampsia are thought to be due to the breakdown of this immune tolerance (6, 9, 10). In pre-eclampsia, whilst the clinical manifestations such as hypertension and proteinuria are thought to be due to endotheliopathy secondary to insufficient placentation (11, 12), the shallow fetal trophoblast invasion is likely related to partial breakdown of maternal–fetal immune tolerance (9).

In this review, we will explore the role of decidual innate and the adaptive immune cells in facilitating tolerance to the fetus. In particular, we will highlight some of the recent advances documenting the interaction between these cells, drawing comparisons between healthy human pregnancy and pre-eclampsia.

## Innate Immune Cells at the Fetal–Maternal Interface

### Decidual antigen presenting cells during pregnancy

Antigen presenting cells are likely to be important players in the mediation of immune tolerance in the decidua. In mice, a previous study has shown that maternal APCs take up apoptotic debris from the fetal/placental cells and present fetal antigens to maternal T cells. As the major histocompatibility antigens (classical MHC I and II antigens) are suppressed on fetal trophoblast cells to evade maternal immune recognition, antigen presentation of fetal minor histocompatibility antigens by maternal APCs is an important route for immune recognition (7). Therefore, exploring the characteristics of the decidual APCs and their interaction with decidual T cells is of great importance in the understanding of fetal–maternal immune tolerance.

### Decidual dendritic cells in healthy pregnancy and pre-eclampsia

Study of decidual dendritic cells (dDCs) has been difficult, not only because isolation of decidual cells including dDCs can be technically demanding, but also because phenotypic definition of DCs is controversial as there is no single specific marker for DCs. In this particular section, we refer primarily to the lineage negative HLA-DR^+^ classical DCs. Using lineage negative and HLA-DR^+^ as combination marker for dendritic cell (DC), Gardener et al. found that dDC comprises ~1% of the total decidual cell isolates in first trimester decidua (13). These DCs were CD11c^+^, CD1a^−^, and CD123^−^, indicating a myeloid rather than plasmacytoid origin. Interestingly, they showed that these DCs were DC-SIGN^−^, compared to CD14^+^ “macrophages,” which were DC-SIGN^+^. These results were further explored in a later study by Ban et al., who showed that first trimester lineage negative and HLA-DR^+^ dDCs predominantly expressed BDCA1 and BDCA3 surface antigens, corresponding with different subsets of myeloid DCs (14).

Overall, due to the difficulty in decidual mononuclear cell isolation and the rarity of dDCs, functional studies on these DCs are scarce. In a study by Kammerer et al., the authors demonstrated a small population of mature CD83^+^ DCs as well as CD1a^+^ DCs in human first trimester decidua, by both immunohistochemistry and flow cytometry (15). They went on to show that the CD83^+^ cells are potent stimulators in mixed lymphocyte reactions comparable to mature peripheral blood monocyte-derived DCs. Another study by Laskarin et al. showed that CD1a^+^ DC isolated from decidua stimulated NK cell activity and proliferation better than decidual CD83^+^ DCs (16). Their experiments were done *in vitro*, however, and there was no demonstration of CD1a^+^ or CD83^+^ DC interaction with decidual NK cells *in situ*. An earlier study demonstrated that lineage− and HLA-DR^+^ DCs in first trimester human decidua were mostly of myeloid origin, but produced less IL-12 compared to their peripheral counterparts. They also showed that dDCs were more likely to prime CD4 cells into a Th2 phenotype compared to their peripheral counterparts (17). The authors concluded that such polarization of the immune response toward Th2 has potential roles in averting Th1-mediated rejection of the fetus.

Studies of decidual DC functions in mice are more definitive. Selective ablation of CD11c^+^ decidual DCs leads to failure of decidualization and embryo implantation (18), highlighting the potential role of dDCs in the initiation of successful pregnancy. Another study demonstrated that during murine pregnancy, decidual CD11c^+^ DCs fail to migrate to draining lymph nodes due to absent lymphatic vessels and CCL21 (ligand for lymphoid homing CCR7) expression in the murine decidua and therefore do not significantly contribute to anti-fetal T cell responses (19). However, it is important to note that in contrast to mice, lymphatic vessels are abundant and CCL21 is expressed within the human decidua (20, 21), which therefore might facilitate decidual DC migration in humans. Furthermore, whilst these studies shed light on the function of CD11c^+^ DCs in mice, it is difficult to know whether these CD11c^+^ cells are comparable to the lineage negative, HLA-DR^+^CD11c^+^ DCs in human decidua. Nevertheless, at least in mice, decidual CD11c^+^ DCs appear to be important for the initiation of pregnancy and maintenance of immune tolerance.

So far, few studies have examined the role of decidual DCs in pre-eclampsia. Huang et al. found that there were increased numbers of CD83^+^ and DC-SIGN^+^ APCs in the pre-eclamptic decidua (22). Scholz et al. partially confirmed this finding showing increased numbers of DC-SIGN^+^ cells in the decidua of patients affected by HELLP syndrome, a severe form of pre-eclampsia (23). However, it is important to note that DC-SIGN^+^ APCs in particular, are likely a different group of cells distinct from lineage negative HLA-DR^+^CD11c^+^ classical myeloid DCs (discussed above), as highlighted in subsequent sections.

### Decidual macrophages in healthy pregnancy and pre-eclampsia

Macrophages are specialized phagocytic cells of the innate immune system and they are present in every organ of the body in one form or another. Macrophages, like DCs, are part of the mononuclear phagocyte system consisting of committed bone marrow precursors, peripheral blood monocytes and DCs, as well as tissue macrophages and DCs (24). Whilst many have attempted to separate macrophages from DCs based on phenotype and function, significant controversy exists as to whether these cells are indeed distinct from one another (25).

CD14^+^ decidual macrophages (dMacs) comprise about 10–20% of decidual CD45^+^ leukocyte population (26). Their phenotype has been characterized in several studies. In a study of human CD14^+^ dMacs, Heikinnen et al. (27) observed that compared to the peripheral blood monocytes, dMacs expressed lower level of co-stimulatory molecule CD86. This coupled with the expression of indoleamine 2,3-dioxygenase (IDO), known to have an immunosuppressive effect on T cells, led them to conclude that dMacs have an “immunosuppressive” phenotype. Notably however, their data showed that dMacs expressed higher level of HLA-DR, as well as the co-stimulatory molecule CD80, compared to peripheral blood monocytes. Another study by Repnik et al. (28) confirmed the expression of HLA-DR, CD80, and CD86 on dMacs. They further showed that expression of these markers were higher earlier in the gestation, implying greater dMac activation at the time of implantation. A more recent study examined dMac in the first trimester using gene micro-array analysis. The authors found that compared to peripheral blood macrophages, dMacs have a gene expression profile, which biases toward alternatively activated macrophages or M2 phenotype, which suggests that dMacs are likely immunosuppressive (29).

In a study of dMac function, Mizuno et al. (30) showed that dMacs have antigen presentation capacity, but are less stimulatory and produce less IL-1 than peripheral blood monocytes in mixed lymphocyte reactions. The suppressive activity of dMac has also been supported by other studies (31). The cytokine profile of dMac was also examined by Heikkinen et al., showing that term decidual CD14^+^ dMac spontaneously produced significantly more IL-10 than peripheral blood monocytes *ex vivo*. In addition, these macrophages were less able to differentiate into mature DCs *in vitro* under polarizing conditions, possibly owing to their production of IL-10 (27). Thus, it is likely that dMacs are a special subset of APCs specialized in tolerance induction. In addition, there is evidence that dMacs are also involved in vascular remodeling (5, 32) and parturition in the peripartum period (33, 34).

In a large study with 33 pre-eclamptic patients and 66 controls, Rieger et al. examined decidual leukocyte populations using flow cytometry (35). They did not find any difference in HLA-DR, dendritic cell specific intercellular adhesion molecule 3 (ICAM3) grabbing non-integrin (DC-SIGN), or CD14 expression within CD45^+^ cells between healthy pregnancy and pre-eclampsia. In a smaller study, Schonkeren et al. compared the distribution and phenotype of CD14^+^ dMacs between preterm control pregnancies and preterm pre-eclampsia (36). Using sequential or two-color immunohistochemistry, they found reduced CD163/CD14 ratio [CD163 being a marker of alternatively activated macrophage or M2 (37)], increased DC-SIGN/CD14 ratio, and reduced IL-10 expression in preterm pre-eclamptic pregnancies, which may suggest a more pro-inflammatory phenotype of dMacs in pre-eclampsia. More recently, we examined decidual CD14^+^ APCs in more detail during healthy pregnancy and pre-eclampsia using multi-color flow cytometry (38). However, in this study, we focused on the distinct subset of CD14^+^DC-SIGN^+^ APCs, which is discussed below.

### Decidual CD14^+^DC-SIGN^+^ APCs in healthy pregnancy and pre-eclampsia

Dendritic cell specific ICAM3 grabbing non-integrin is an ICAM3 receptor, where ICAM3 is an adhesion molecule. DC-SIGN, also known as CD209, is important for the initiation of DC and T cell interaction (39). Despite its name, DC-SIGN may be expressed by a variety of APCs other than classical lineage negative HLA-DR^+^ DCs, including CD14^+^ macrophages (40). Nevertheless, in monocyte-derived DCs, DC-SIGN is one of the markers upregulated in maturing DCs in mice (33) and humans (39). Therefore, whilst the expression of DC-SIGN is not DC specific, it probably marks myeloid cells, which are on the DC differentiation pathway (i.e., immature DCs).

In the human decidua, Kammerer et al. found that a significant percentage of CD14^+^HLA-DR^+^ APCs expressed DC-SIGN in the first trimester decidua (41). These CD14^+^DC-SIGN^+^ cells did not express CD83, but expressed CD4. Interestingly, the authors found these cells to be unique to the decidua in pregnancy and not in normal non-pregnant endometrium. In addition, these cells show a high proliferative rate and good antigen uptake, but poor stimulatory activity in MLR. Importantly, these cells have a veiled appearance typical of immature DCs on immunohistochemistry and can be matured *in vitro* with a cocktail of inflammatory cytokines into CD83^+^ mature DCs, with decreased CD14 and DC-SIGN expression, as well as potent stimulatory activity in MLR. The authors concluded that these CD14^+^DC-SIGN^+^ cells are likely to be precursors of DCs and may play an important role in mediating fetal–maternal immune tolerance. Repnik et al. also confirmed DC-SIGN expression in decidual CD14^+^ APCs and showed that DC-SIGN expression peaked in the second trimester (28). Obviously, decidual CD14^+^DC-SIGN^+^ APCs would be included in studies examining dMacs in view of their CD14 expression. Such studies include recent work by Svensson et al., who showed that CD14^+^ dMacs can be divided into two distinct groups based on ICAM3 expression, with the ICAM3^−^ group expressing DC-SIGN and markers of alternative (M2) macrophage activation (CD163, CD206, neuropilin) (42). They further showed that the phenotype of these DC-SIGN^+^ dMacs may be replicated *in vitro* (with similar gene expression profile) in the presence of M-CSF (and/or GM-CSF) plus IL-10. Another study divided first trimester CD14^+^ dMacs into CD11c^hi^ and CD11c^lo^ cells corresponding to DC-SIGN^−^ and DC-SIGN^+^ cells, respectively (43). Using gene expression profiles, the authors here showed that neither of the CD11c^hi^ or CD11c^lo^ macrophages corresponds to *in vitro* differentiated M1 or M2 macrophages exactly, though CD11c^hi^ macrophages were skewed toward maternal peripheral blood monocytes and shared common genes with synovial macrophages from rheumatoid arthritis patients. In the same study, the authors showed that CD11c^hi^ dMac produced significantly more TNFα, IL-6, and paradoxically IL-10 compared to CD11c^lo^ macrophages. On the other hand, there was a slight trend toward increased TGFβ secretion by CD11c^lo^ cells.

Collectively, these studies confirm that the human decidua harbors two distinct populations of CD14^+^ APCs, one which is CD11c^lo^DC-SIGN^+^CD206^+^CD163^+^neuropilin^+^ICAM3^−^ and is likely immunoregulatory and important for tolerance induction, the other which is CD11c^hi^DC-SIGN^−^CD206^−^CD163^−^ neuropilin^−^ ICAM3^+^ and probably pro-inflammatory and important for tissue remodeling. In our recent study, we examined term decidual CD14^+^DC-SIGN^+^ APCs in detail using multi-color flow cytometry. We show that decidual CD14^+^DC-SIGN^+^ APCs expressed significantly higher amount of tolerogenic molecules (HLA-G and ILT4), lymphoid homing molecule (CCR7), as well as antigen presentation apparatus (HLA-DR, CD80, CD86), but less CD14 than CD14^+^DC-SIGN^−^ cells (38). This suggests that decidual CD14^+^DC-SIGN^+^ APCs may be further along the differentiation pathway than their DC-SIGN^−^ counterparts and that these cells possess enhanced tolerogenic properties. Both of these observations are consistent with the previously described studies (42, 43). The tolerogenic properties are likely induced by IL-10, which is known to upregulate HLA-G and ILT4 (44, 45). Interestingly *in vitro*, we were able to differentiate peripheral blood monocytes into CD14^+^DC-SIGN^+^HLA-G^+^ILT4^+^ APCs by adding IL-10 to the DC polarizing protocol (with GM-CSF and IL-4) (46). In the context of the blurred border between macrophages and DCs, and given that the phenotype of decidual DC-SIGN^+^ APCs may be replicated *in vitro* with both DC or macrophage polarizing protocols, we suggest that decidual CD14^+^DC-SIGN^+^ APCs are likely an intermediate cell type on the continuum of macrophage/DC differentiation under the influence of IL-10. Whether decidual CD14^+^DC-SIGN^−^ APCs are completely distinct from, or on the same developmental continuum as, CD14^+^DC-SIGN^+^ APCs is currently unknown. However, the later hypothesis is supported by the fact that CD14^+^DC-SIGN^−^ APCs are closer to peripheral blood monocytes (43) and possess less antigen presentation apparatus. The differences between decidual CD14^+^DC-SIGN^+^ and CD14^+^DC-SIGN^−^ APCs are summarized in Table 1.

**Table 1:** **Differences between decidual DC-SIGN^+^ and DC-SIGN^−^ APCs**.

	DC-SIGN^+^	DC-SIGN^−^
**LINEAGE MARKER**		
CD14 (38)	Intermediate	High
CD4 (41)	Intermediate	Low
**ANTIGEN PRESENTATION APPARATUS (38)**		
HLA-DR	High	Intermediate
CD80	High	Low
CD86	High	Intermediate
**ADHESION MOLECULES**		
CD11c (43)	Low	High
ICAM3 (42)	Low	High
CCR7 (38)	High	Low
**M2 MARKERS (42)**		
CD163	High	Low
Neuropilin	High	Low
**TOLEROGENIC MOLECULES (38)**		
HLA-G	High	Low
ILT4	High	Intermediate
**CYTOKINE PRODUCTION (43)**		
TNFα, IL-6	Low	High
IL-10	Low	High
TGFβ	High	Intermediate
% CD14+ cells (first trimester) (43)	~70%	20–30%
*In vitro* differentiation	M-CSF ± GM-CSF + IL-10 (42); GM-CSF+IL-4+IL-10 (46)	Unknown
*In vitro* differentiation to DC (41)	Yes	Probably
Likely function	Immune regulation	Tissue remodeling

Interestingly in pre-eclampsia, we found an increased percentage of DC-SIGN^+^ APCs within the CD14^+^ population, however, pre-eclamptic decidual CD14^+^DC-SIGN^+^ APCs expressed significantly less HLA-G and ILT4 compared to the same cells in healthy pregnancy, suggestive of reduced tolerogenic capacity. We speculate that this phenotypic difference may be related to the reduced placental IL-10 levels in pre-eclamptic pregnancies (47).

**Figure 1:**
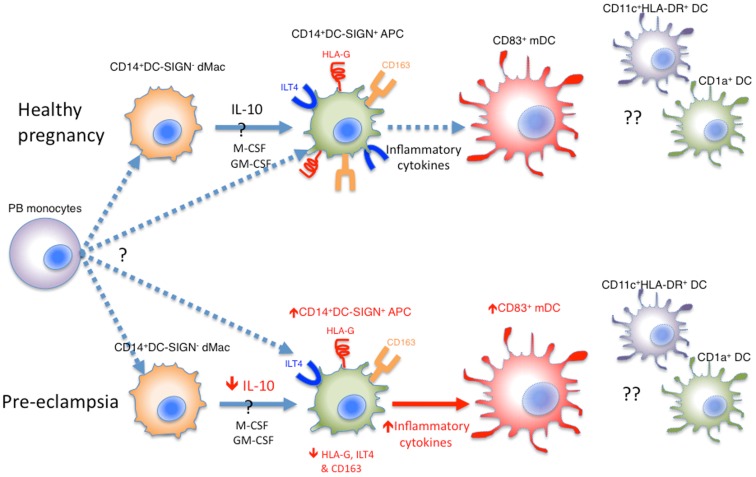
**Various APCs in the human decidua**. Whether decidual CD14^+^DC-SIGN^−^ dMacs and CD14^+^DC-SIGN^+^ APCs are derived independently from peripheral blood (PB) monocytes is unknown. Alternatively, CD14^+^DC-SIGN^−^ dMacs may be on a continuum of DC differentiation, where local IL-10, M-CSF, and GM-CSF drive their development into CD14^+^DC-SIGN^+^ APCs expressing HLA-G and ILT4. These APCs may be matured into CD83^+^ mature DCs (mDCs) under the influence of inflammatory cytokines, which in healthy pregnancy is minimal. In pre-eclampsia, both CD14^+^DC-SIGN^+^ APCs and mDCs increase, probably driven by increased inflammatory cytokines in this disease. This is coupled with reduced HLA-G and ILT4 expression by CD14^+^DC-SIGN^+^ APCs, likely due to reduced local IL-10 levels. Whilst CD11c^+^HLA-DR^+^ and CD1a^+^ DCs have been identified in human decidua, their roles and relationship with CD83^+^ DCs are unclear.

In summary, there are several different types of APCs present in the decidua, including lineage negative HLA-DR^+^CD11c^+^ classical DCs, mature CD83^+^ DCs, CD1a^+^ DCs, and CD14^+^DC-SIGN^−^ dMac, which may have developed from peripheral blood monocytes and are probably precursors to decidual CD14^+^DC-SIGN^+^ APCs. Their potential relationships and differences in healthy pregnancy and pre-eclampsia are summarized in Figure 1.

### Decidual NK cells in healthy pregnancy and pre-eclampsia

Decidual NK cells (dNK) are the most abundant maternal leukocytes in the decidua, especially in the first trimester, making up 70% of the maternal CD45^+^ leukocyte population. (48) The dNK cells are distinct from majority of peripheral blood NK cells, in that they are large, granular, and are CD56^hi^ and CD16^−^ (8). The origin of these cells is unclear, although some have proposed possible recruitment of a subset of peripheral blood CD56^hi^ NK cells into the decidua (49). Interestingly, during early pregnancy, dNK accumulate as a dense infiltrate around the trophoblast cells, but they progressively decrease in number from mid-gestation onward (50). This timing seems to implicate that dNK cells may be involved in modulating trophoblast invasion and vascular remodeling. Indeed, dNK have been shown to produce vascular endothelial growth factor C (VEGFC), placental growth factor (PIGF), and angiopoietin 2 (ANG2).

Decidual NK cells may also be important in modulating the degree of trophoblast invasion, as they are seen in close proximity to the invading trophoblasts in the decidua. Certainly, dNK have been shown to express killer inhibitory receptor (KIR) (51), CD94/NKG2A (52), and ILT2 (53), which recognize HLA-C and HLA-G, respectively, expressed on trophoblast cells. The HLA-C–KIR interaction is thought to be important in the pathogenesis of pre-eclampsia. As HLA-C is dimorphic and KIR polymorphic, it has been shown that certain combinations of maternal KIR and fetal HLA-C lead to an increased risk of pre-eclampsia, possibly through modulation of trophoblast migration, implying that HLA-C–KIR interaction is important in placentation (54, 55). However, NK cell KIR and HLA-C mismatch clearly does not explain all cases of pre-eclampsia, since only 30% of pre-eclamptic pregnancies have the at-risk maternal KIR phenotype (KIR AA) (54). HLA-G–ILT2 interaction on NK cells on the other hand, has been shown to increase dNK secretion of inflammatory and proangiogenic factors such as IL-1β, IL-6, TNF, and IL-8 (56). NK cells themselves are also susceptible to modulation by the decidual cytokine milieu. Indeed, dNK cells are thought to be the mediator of fetal demise in IL-10-deficient mice treated with LPS, which conversely can be rescued by administration of IL-10 (57). This suggests that IL-10 may modulate dNK cell cytotoxicity.

Collectively, these data suggest that dNK cells are important for modulation of trophoblast invasion and decidual vascularization in pregnancy. However, the recognition and tolerance of paternal allo-antigens via APC presentation during pregnancy, clearly requires the participation of other limbs of the immune system, such as the adaptive immune cells.

## Adaptive Immune Cells at the Fetal–Maternal Interface

The adaptive immune system distinguishes itself from the innate immune system by its antigen specificity and immunological memory. Therefore, the fact that pre-eclampsia is essentially a disease of primigravida and subsequent pregnancies with the same partner protect against pre-eclampsia (58, 59), supports the involvement of the adaptive immune system. Within the lymphocyte subsets, CD4^+^Foxp3^+^ regulatory T (Treg) cells in particular have been the subject of many studies, with their pivotal role in pregnancy now firmly established. Th17 cells, the pro-inflammatory antagonist of Treg cells have also become a focus of studies in the last few years. More recently, CD4^+^HLA-G^+^ suppressor T cells have also been implicated for their potential role in healthy pregnancy and pre-eclampsia. Whilst other cells within the adaptive immune system such as Th1, Th2, gamma-delta T cells, and CD8^+^ T cells also play a role in fetal–maternal immune tolerance (60–63), our focus in this review will be on Treg, Th17, and CD4^+^HLA-G^+^ suppressor T cells.

### Treg cells in healthy pregnancy and pre-eclampsia

Foxp3^+^ Treg cells are a unique subset of suppressive CD4^+^ T helper cells indispensable for immune tolerance to self- and foreign-antigens in humans and mice (64–67). Several authors have shown that in pregnancy, there is an expansion of peripheral blood Treg cell pool in both humans (68, 69) and mice (70). The study by Somerset et al. showed that Treg cell population seems to peak in the second trimester and thereafter decreases to slightly above normal levels at delivery of the conceptus. Some have suggested that this expansion of Treg cell is not allo-antigen driven at least in the mice, as both syngeneically and allogeneically pregnant mice show expansion of the Treg cell population (70). However, the authors did not show a direct comparison of Treg cell percentage between syngeneic and allogeneic pregnancies. In contrast, Zhao et al. showed through direct comparison, that the percentage of peripheral blood Treg cells in allogeneic pregnancy is higher compared to syngeneic pregnancy, suggesting that the expansion of Treg cell is at least partially allo-antigen driven (71). Since then, other studies have demonstrated the fetal antigen-specific nature of maternal Treg cells during pregnancy (72, 73), further supporting the role of fetal allo-antigen in Treg cell expansion. Therefore, whilst other factors such as pregnancy-related hormones can also contribute to Treg cell expansion (74–76), it is likely that fetal allo-antigen stimulation is the primary driving force.

Given the decidua is the fetal–maternal interface and the likely place of fetal antigen encounter, it is not surprising that the proportion of Treg cells is even greater in the decidua during pregnancy compared to the peripheral blood (77, 78). The question is whether these Treg cells are recruited from the peripheral blood or induced locally. Currently in humans, despite some controversy, the only marker that differentiates thymus-derived natural Treg (nTreg) cells from peripherally induced Treg (iTreg) is Helios, where Helios^+^ Treg cells are nTreg cells, which have acquired Treg cell phenotype in the thymus, whereas Helios^−^ Treg cells have differentiated in the peripheral tissues/lymph nodes from naïve T cells (79). Based on this premise, we found that the proportion of Helios^−^ iTreg cells was significantly higher in the decidua compared to the peripheral blood (38). Interestingly, our results also indicate that the previously described peripheral blood expansion of Treg cells associated with healthy pregnancy is accounted for by the expansion of iTreg cells, and not nTreg cells. This suggests that in healthy pregnancy, iTreg cells are induced locally in the decidua/draining lymph nodes most likely in response to fetal allo-antigens. This observation is consistent with the murine studies previously discussed (72, 73), as well as the fact that iTreg cells are thought to facilitate tolerance to foreign- (in this case fetal) and self-antigens, whereas nTreg cells are primarily involved in self-tolerance (67, 80, 81).

Functionally, *in vivo* experiments in the murine model have shown that Treg cells are important for fetal–maternal immune tolerance. Aluvihare et al. showed that adoptive transfer of whole T cell populations to T cell-deficient pregnant mice did not result in fetal rejection, whereas transfer of T cells depleted of CD25^+^ Treg cells led to fetal demise, especially in allogeneic pregnancies (70). This was confirmed by another method, where PC61 monoclonal antibody against CD25 was used to deplete Tregs in murine syngeneic and allogeneic pregnancy. The authors found that fewer fetuses in allogeneic pregnancies survived to term whereas syngeneic pregnancies were not affected by Treg cell depletion (82). This indicates that Treg cells are critically required for allogeneic but not syngeneic pregnancy.

Whereas total Treg cell depletion leads to almost complete fetal rejection (70, 82), depletion of the iTreg, but not nTreg, compartment in CNS1 (conserved non-coding sequence 1) deficient mice (CNS1 being critical for iTreg development) leads to partial fetal resorption (~10%) and abnormal spiral artery formation in allogeneic murine pregnancies (83). This phenotype is reminiscent of the human disease – pre-eclampsia, where IUGR (intrauterine growth retardation) and abnormal spiral artery remodeling with shallow placentation is a key pathological feature (84), being mindful of the caveat that there are significant differences between human and murine pregnancies (85).

Nevertheless, our investigations did show that the blunted peripheral blood Treg cell expansion in pre-eclampsia (69, 86, 87) is primarily due to the failure of iTreg cell expansion, whereas nTreg cells are not affected. Interestingly in the pre-eclamptic decidua, whilst we did not find a reduction in total Treg cell percentage, there was a significant reduction in the percentage of Helios^−^ iTreg cells compared to healthy pregnancy (38). These observations suggest that in pre-eclampsia, there is impaired expansion of iTreg cells in the decidua, with compensatory nTreg cell recruitment to avert fetal rejection. Collectively, these data from murine and human studies suggest that iTreg and nTreg cells collaborate to maintain fetal–maternal immune tolerance, such that complete lack of Treg cells leads to fetal rejection, whereas specific iTreg cell deficiency results in poor placentation and fetal growth restriction.

### Th17 cells in healthy pregnancy and pre-eclampsia

Th17 cells are a subset of CD4^+^ T helper cells, which secrete the pro-inflammatory cytokines IL-17, IL-22, regulated by the transcription factor RORγt (88). Importantly, both iTreg and Th17 cells are derived from naïve CD4^+^ T cells under the influence of TGFβ (89) in a concentration-dependent manner, with high levels of TGFβ favoring iTreg cell induction and low concentrations favoring development of Th17 cells (88, 90). Additionally, the presence of the pro-inflammatory cytokine, IL-6 is crucial for skewing T cell differentiation toward Th17 phenotype (91).

**Figure 2:**
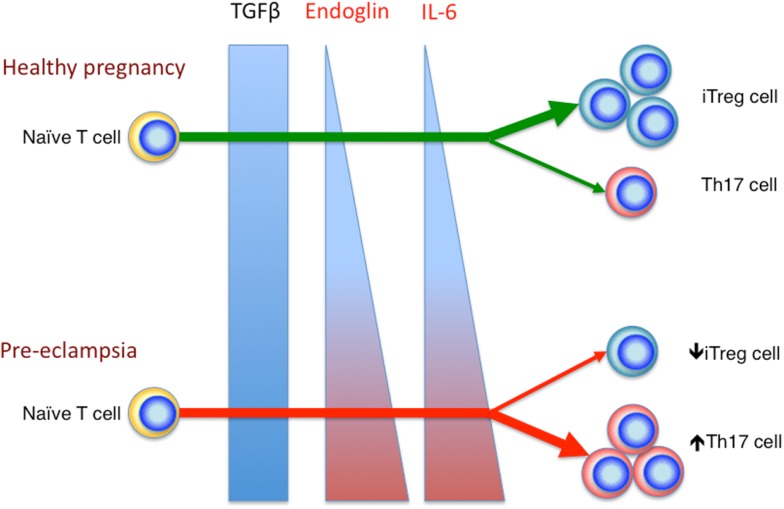
**Reciprocal development of iTreg and Th17 cells in healthy pregnancy and pre-eclampsia**. In the relative absence of IL-6 and endoglin, TGFβ signaling enhances iTreg, but not Th17 cell differentiation in healthy pregnancy. In pre-eclampsia, the elevated level of endoglin could reduce TGFβ signaling, which in combination with the increased IL-6 level, would deter iTreg cell induction, but drive Th17 differentiation.

We have previously shown that the percentage of peripheral blood Th17 cells decreases in healthy pregnancy, in stark contrast to the expanding iTreg cell population. Whereas in pre-eclampsia, the percentages of peripheral blood Th17 and iTreg cell subsets remain comparable to the non-pregnant state. This leads to an increased Treg: Th17 ratio in healthy pregnancy, which is blunted in pre-eclampsia (69). These results were later replicated in other studies (92, 93). Collectively, these observations are congruous with the raised serum IL-6 level in pre-eclampsia compared to healthy pregnancy (94), which may contribute to the increased Th17 cells compared to normal pregnancy. Additionally soluble endoglin, a circulating TGFβ glycoprotein receptor capable of inhibiting TGFβ signaling, is elevated in pre-eclampsia (95). This would likely reduce the degree of TGFβ signaling, hence favoring Th17 cell differentiation. Therefore, current evidence indicates that in healthy pregnancy, there is a preferential differentiation of iTreg cell over Th17 cells, which is deranged in pre-eclampsia, possibly related to altered systemic levels of various factors such as IL-6 and soluble endoglin (Figure 2).

Currently, few studies have examined Th17 cells in the human decidua. A study by Mjosberg et al. attempted to examine the proportions of Th1, Th2, Treg, and Th17 cells in early pregnancy decidua, however, in this study, the authors used chemokine receptors as surrogate markers for Th1, 2, and 17 cells, which is less than ideal, they concluded that there is near absence of Th17 cells in early healthy pregnancy decidua (96). Wang et al. found increased peripheral blood and decidual Th17 cell percentage in women with unexplained recurrent miscarriages compared to healthy pregnancy. In their samples, the percentage of Th17 cells in the decidua appears to be comparable or even slightly lower than in peripheral blood in both study groups (97). This is in contrast to another study, which showed higher percentages of Th17 cells in the decidua in healthy pregnancy (98). Therefore, current evidence demonstrates that Th17 cells are present in the decidua, although their prevalence is controversial. Furthermore, no study to date has examined the presence and prevalence of decidual Th17 cells in pre-eclampsia. It would be interesting to see whether there is an increase in decidual Th17 cells in pre-eclampsia, which would add to the body of evidence implicating local immune dysregulation in this disease.

### CD4^+^HLA-G^+^ T cells in healthy pregnancy and pre-eclampsia

HLA-G is an atypical MHC class I molecule first discovered on human trophoblasts (99, 100). It exerts immunosuppressive effects on various immune cells, including APCs, NK cells, and T cells (101–103). Therefore, it is not surprising that it is expressed at immune privileged sites such as the decidua (99), the cornea (104), and thymic medulla (105). Interestingly, distinct subsets of HLA-G^+^ T cells (both CD4^+^ and CD8^+^) are present at low levels in the peripheral blood of healthy donors (106). These cells are immunosuppressive but do not express Foxp3. They mediate suppression in a HLA-G and IL-10-dependent manner (107). Some evidence suggests that these cells originate from the thymus (106), however others have also shown that activated T cells can also “acquire” HLA-G from HLA-G expressing APCs, through the process of trogocytosis (108). Trogocytosis is a process by which membrane fragments including surface molecules are transferred from one cell to another in a contact-dependent manner (109). These HLA-G expressing T cells are similarly immunosuppressive (108, 110).

We and others have shown that the percentage of peripheral blood CD4^+^HLA-G^+^ T cells is significantly increased in healthy pregnancy, which is even more pronounced within the decidua, where up to 20% of the CD4^+^ T cells are HLA-G^+^ (46, 111). These CD4^+^HLA-G^+^ T cells are more mature and activated than their HLA-G^−^ counterparts, they are Foxp3^−^ but are immunosuppressive (46). Importantly, in pre-eclampsia there is impaired expansion of these CD4^+^HLA-G^+^ T cells in both the peripheral blood and the decidua. This is in keeping with the reduced serum HLA-G and placental HLA-G level in this disease, and reinforces the dysregulated adaptive immune responses in pre-eclampsia.

## Innate and Adaptive Interaction at the Fetal–Maternal Interface

The evidence and discussions presented so far have focused on individual cell populations and their role in fetal–maternal immune tolerance, however, the immune system clearly does not work in isolation, but rather like an intricate, changing network. It is therefore important to investigate the interactions between the various immune cells, whether innate or adaptive at the fetal–maternal interface. In the following sections of this review, we will focus on the current available evidence in this regard and attempt to present a unifying concept, as well as future research directions for innate and adaptive immune interactions within the decidua.

### Decidual NK cell crosstalk with innate and adaptive immune cells

As discussed previously, dNK cells are important for modulating fetal trophoblast invasion and vascular remodeling. However, emerging evidence also suggests that dNK cells interact and modulate other maternal immune cells. Kammerer et al. first noted that dNK cells are closely associated with decidual DC-SIGN^+^ APCs. They further demonstrated that this interaction occurs through ICAM3 (expressed on NK cells) and DC-SIGN interaction, although it was unclear what this interaction involves (41).

A later study demonstrated that dNK cells modulate decidual CD14^+^ macrophages (dMac) to expand Treg cells *in vitro* (112). In this study, Vacca et al. demonstrated that interaction between dMac and dNK cells led to release of IFNγ by dNK cells, the IFNγ in turn induces upregulation of IDO in dMac. Importantly, IDO is known to contribute to immune suppression at the fetal–maternal interface (113, 114). It works by catabolizing tryptophan into l-kynurenine, which results in impaired T cell activation and favors Treg cell induction (115). Indeed, Vacca et al. showed that the IDO induction was important for subsequent Treg cell expansion by dMacs cultured with dNK, along with other factors such as TGFβ and CTLA-4 engagement. Notably, CTLA-4 engagement of APC co-stimulatory B7 molecules has also been shown to upregulate IDO expression in APCs (116). This could provide a continuous positive reinforcement loop, where the expanded CTLA-4 expressing Treg cells further enhance APC IDO expression. Interestingly, l-kynurenine inhibited the ability of peripheral blood NK cells, but not dNK cells to secrete IFNγ, which may explain how this negative feedback prevents peripheral blood NK cells from modulating dMacs in the same way. Although the authors did not clarify, these “dMacs” are probably decidual DC-SIGN^+^ APCs, which interact with ICAM3^+^ dNK cells via DC-SIGN. It is also important to note that the authors did not clearly demonstrate *de novo* Treg cell induction under these conditions, since the starting peripheral blood CD3^+^ population would contain nTreg cell population. Nevertheless, the interaction between dNK and dMac appears to favor Treg cell proliferation and expansion through IFNγ-induced upregulation of IDO.

Interestingly, a more recent study showed that dNK production of IFNγ may be important for averting Th17 differentiation at the fetal–maternal interface (55). Here, the authors found that CD56^hi^CD27^+^ dNK cells are particularly primed to secrete IFNγ, which has been shown to inhibit Th17 differentiation via STAT1 activation (117). They went on to show that in the murine model, deletion of NK cells led to increased Th17 cell accumulation in the decidua and increased fetal loss. *In vitro*, dNK cell-derived IFNγ significantly inhibited Th17 cell differentiation, an effect that was reversed by IFNγ neutralizing antibodies. Finally they showed that there is reduced CD56^hi^CD27^+^ dNK cell:Th17 cell ratio in the decidua of women with recurrent miscarriages, accompanied by reduced IFNγ secretion by dNK cells in these women. These results suggest that a special subset of CD56^hi^CD27^+^ dNK cells may be important for limiting Th17 cell differentiation and inflammation in normal pregnancy via IFNγ. It is intriguing to contemplate whether this inhibition of Th17 cell differentiation may paradoxically promote iTreg cell development, since both develop along the same pathway and reciprocally inhibit one another (84, 118). Furthermore, whether similar, but perhaps milder, pathophysiology may be found in pre-eclampsia is also of interest and requires further investigations.

Thus, these recent results highlight that NK cells are able to influence both decidual APCs and T cells through their secretion of IFNγ to promote immune tolerance.

### APC and T cell interaction at the fetal–maternal interface

Antigen presenting cells play important roles in shaping T cell responses and differentiation; T cells in turn also modulate APC function. In our recent study, we showed by immunohistochemistry that decidual DC-SIGN^+^ APCs are closely associated with Foxp3^+^ Treg cells (38). In fact the number of DC-SIGN^+^ APCs correlated significantly with Foxp3^+^ Treg cells in healthy pregnancy, but interestingly not in pre-eclampsia, suggesting a dysregulated relationship between these cells in this disease. We went on to show that decidual CD14^+^DC-SIGN^+^ APCs from healthy pregnant, but not pre-eclamptic women induced iTreg cells significantly more efficiently than CD14^+^DC-SIGN^−^ APCs. This suggests that there is an intrinsic defect in decidual CD14^+^DC-SIGN^+^ APCs in pre-eclampsia. This is consistent with the reduced expression of the tolerogenic molecules HLA-G and ILT4 in this cell subset in pre-eclampsia. These results are also consistent with Vacca et al.’s study described in the previous section (112), and reinforce that decidual DC-SIGN^+^ APCs are an important population of cells in human pregnancy, which likely present fetal allo-antigens and induce local iTreg cells. Importantly, in contrast to CD14^+^DC-SIGN^−^ APCs, decidual CD14^+^DC-SIGN^+^ APCs express CCR7 (38), which suggests that they may also migrate to uterine draining lymph nodes and induce iTreg cells there.

The unresolved question is how these CD14^+^DC-SIGN^+^ APCs induce iTreg cells. Vacca et al.’s study suggests that TGFβ and IDO may be required for this process (112). This raises another intriguing question, as to whether the elevated endoglin levels in pre-eclampsia may impair TGFβ signaling and impede iTreg cell induction whilst promoting Th17 differentiation. It is important to note however, that our experiments were done *in vitro*, removed from the *in vivo* environment where endoglin may play a role. Therefore, our data indicates that there may be an intrinsic defect in CD14^+^DC-SIGN^+^ APCs in pre-eclampsia. Perhaps in pre-eclampsia, decidual CD14^+^DC-SIGN^+^ APCs secrete less TGFβ, or have reduced IDO expression?

The abundance of immunosuppressive CD4^+^HLA-G^+^ T cells in the decidua raises the question regarding how these cells have developed. Since HLA-G could be transferred from cell to cell via trogocytosis (108, 110, 119), we reasoned that these CD4^+^ T cells could have acquired HLA-G from any of the HLA-G expressing cells in the decidua, including fetal EVT and maternal CD14^+^DC-SIGN^+^ APCs. To this end, we showed that *in vitro*, decidual CD14^+^DC-SIGN^+^ APCs, but not JEG3 cells (an EVT-like cell line), were able to induce CD4^+^HLA-G^+^ T cells from naïve T cells (46). This is consistent with the fact that T cell trogocytosis is facilitated by the formation of immunological synapse with TCR–MHC engagement (120–122), since EVTs and JEG3 cells do not express MHC II. We further showed that the acquisition of HLA-G from T cells occurred via trogocytosis, since PE labeled HLA-G was passed from the APC to responding T cells. Therefore, we suggest that in the human decidua, T cells activated by fetal allo-antigens may be “silenced” by their acquisition of HLA-G, indeed the HLA-G expressing T cells in the decidua exhibit an activated phenotype (46). Importantly in pre-eclampsia, there is significant reduction of CD4^+^HLA-G^+^ T cells, which may be secondary to the reduced HLA-G expression by decidual CD14^+^DC-SIGN^+^ APCs in this disease.

Based on these recent data, it appears that decidual CD14^+^DC-SIGN^+^ APCs are a unique and important population of cells, which play central roles in regulating local immune responses by their interactions with dNK cells and resident CD4^+^ T cells. As described previously, decidual CD14^+^DC-SIGN^+^ APCs may have developed under the influence of local IL-10 (42, 46), which is probably derived from dNK and CD14^+^DC-SIGN^−^ dMac (41).

Therefore, IL-10 may be a central cytokine driving the differentiation of decidual tolerogenic APCs and suppressor T cells. Interestingly, IL-10 is dispensable for murine pregnancy in the germ free environment, but crucial when LPS is present (57), suggesting that IL-10 is important for controlling inflammation at the fetal–maternal interface. This is not dissimilar to the gut where bacteria colonized, but not germ free mice, with IL-10 deficiency develop severe enterocolitis (123, 124), and humans with defects in IL-10 signaling pathway develop early onset inflammatory bowel disease (125, 126). In the gut, it has been shown that it is the pathogenic T cells that cause disease in the IL-10-deficient mice (81). Furthermore, recent evidence suggests that IL-10 may enhance Treg cell function and augment suppression of pathogenic Th17 cells (127, 128). It remains to be seen whether IL-10 plays similar roles in pregnancy. Nevertheless, it is clear that IL-10 is a crucial regulatory cytokine at mucosal surfaces in the presence of inflammation and foreign antigens. Indeed pre-eclampsia, rather than being an overt rejection of the fetus, is more like a pro-inflammatory state with flaws in the regulatory mechanisms, including reduced IL-10, altered decidual DC-SIGN^+^ APCs, and impaired suppressor T cell (iTreg cells and CD4^+^HLA-G^+^ T cells) differentiation; further fueled by enhanced pro-inflammatory effectors such as raised IL-6 level and enhanced Th17 cell differentiation.

## Concluding Remarks

The fetal–maternal interface is an important frontier for immunology, as it represents the junctional point between two immunologically distinct individuals. Within this mucosal surface, the various maternal innate and adaptive immune cells must work together to ensure tolerance toward invading fetal cells and foreign fetal antigens. Whilst several APCs are present in the decidua, CD14^+^DC-SIGN^+^ APCs are unique at this interface and play central roles in regulating T cell responses, by inducing Foxp3^+^ iTreg cells and CD4^+^HLA-G^+^ suppressor T cells. Some evidence suggests that IL-10 may be an important factor in these processes. Certainly in pre-eclampsia, where there is relative deficiency of IL-10, these tolerance mechanisms are impaired. Whether CD14^+^DC-SIGN^+^ APCs also influence decidual Th17 cell differentiation remains unknown and requires further investigations.

**Figure 3:**
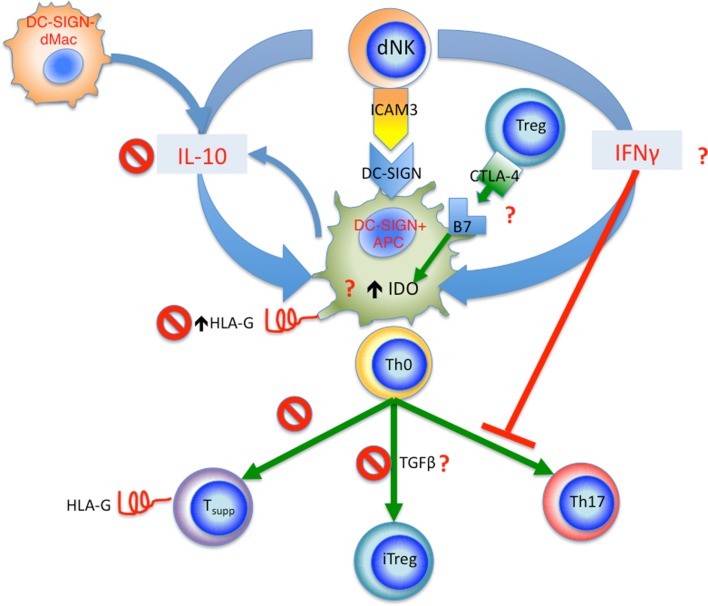
**Summary of proposed innate and adaptive interactions in human pregnancy**. Decidual DC-SIGN^+^ APCs appear to be a central player in these interactions. dNK cells interact with DC-SIGN^+^ APCs via ICAM3, this leads to release of IFNγ, which in turn upregulates IDO in DC-SIGN^+^ APCs, as well as inhibiting Th17 cell differentiation. The tolerogenic DC-SIGN^+^ APCs function to induce iTreg cells from naïve CD4^+^ T cells (Th0), Treg cells in turn regulated DC-SIGN^+^ APCs via CTLA-4 and B7 (CD80 and CD86) interaction, further increasing IDO expression. IL-10 from dNK cells and DC-SIGN^−^ dMacs acts to upregulate HLA-G expression by DC-SIGN^+^ APCs; the HLA-G is then passed onto activated T cells via trogocytosis, resulting in accumulation of CD4^+^HLA-G^+^ T suppressor cells. In pre-eclampsia, several check points are affected as marked by the “No symbol.” These include reduced IL-10 level, reduced HLA-G expression by DC-SIGN^+^ APCs, reduced generation of CD4^+^HLA-G^+^ T suppressor cells, and reduced iTreg cell induction. Other yet unknown mechanisms may also be affected in pre-eclampsia marked by the red question mark, including IFNγ production by dNKs, CTLA-4 expression by Treg cells and interaction with B7 molecules, as well as IDO expression and TGFβ production by DC-SIGN^+^ APCs.

On the other hand, decidual NK cells, on top of their role in modulating trophoblast invasion and vascular remodeling, are able to regulate decidual Th17 differentiation by their production of IFNγ. Interestingly, their production of IFNγ also modulate decidual CD14^+^DC-SIGN^+^ APCs by upregulating their IDO expression to enhance Treg cell expansion. Whether these processes are affected in pre-eclampsia remain to be seen. These interactions are summarized in Figure 3. Clearly, there are likely many other innate and adaptive interactions involving different cell types, which work to foster the delicate balance of immune tolerance at the fetal–maternal interface. Disturbance of the quality and quantity of these interactions likely contribute to the pathogenesis of pre-eclampsia, which like so many diseases of the modern era, is a disease of immune dysregulation.

## Conflict of Interest Statement

The authors declare that the research was conducted in the absence of any commercial or financial relationships that could be construed as a potential conflict of interest.
